# When lesion-directed therapy is not enough: blue rubber bleb nevus syndrome in a young adult—a case report

**DOI:** 10.3389/fped.2026.1916054

**Published:** 2026-09-10

**Authors:** Mingyue Tan, Hao Zhang, Junbin Wang, Ruoyan Ma, Yuncai Zhou, Xuejian Liu

**Affiliations:** 1 The Second Clinical Medicine School, Shandong University of Traditional Chinese Medicine, Jinan, China; 2 Department of Gastrointestinal Surgery, Shandong Provincial Third Hospital, Jinan, China; 3 Department of Emergency Medicine, Shandong Provincial Third Hospital, Jinan, China; 4 The First Rehabilitation Hospital of Shandong, Linyi, Shandong, China

**Keywords:** blue rubber bleb nevus syndrome, case report, gastrointestinal bleeding, multifocal venous malformation, young adult

## Abstract

Blue rubber bleb nevus syndrome (BRBNS) is a rare systemic venous malformation disorder that primarily affects the skin and gastrointestinal tract. We report a 20-year-old Chinese man of Han ethnicity with multifocal cutaneous and soft-tissue venous malformations and recurrent gastrointestinal bleeding since early childhood. Despite repeated endoscopic and surgical interventions, he developed recurrent melena, hematochezia, and severe anemia requiring transfusion. During the current hospitalization, he received packed red-cell transfusion and oral iron supplementation and underwent lauromacrogol sclerotherapy of multiple symptomatic soft-tissue venous malformations. His hemoglobin level increased from 55 to 98 g/L at discharge, and no recurrent gastrointestinal bleeding occurred during 2 months of follow-up. This case illustrates the limitations of repeated lesion-directed therapy in multifocal BRBNS and emphasizes the importance of multidisciplinary management and longitudinal surveillance. Although early clinical and hematologic improvement was observed, longer follow-up is required to determine whether durable disease control has been achieved.

## 1 Introduction

Blue rubber bleb nevus syndrome (BRBNS) is a rare congenital vascular anomaly characterized by multifocal venous malformations that predominantly involve the skin, soft tissues, and gastrointestinal tract (1, 2). Gastrointestinal lesions may cause recurrent occult or overt bleeding, iron-deficiency anemia, and transfusion dependence from childhood into adulthood. Because lesions are often diffuse, increase in number over time, and may recur at sites not amenable to complete resection, long-term management remains challenging. Endoscopic and surgical interventions can control accessible bleeding lesions, but their benefits may be temporary in patients with extensive, multifocal disease (3–5).

The present case documents an 18-year clinical course in a 20-year-old Chinese man of Han ethnicity with cutaneous, soft-tissue, and gastrointestinal venous malformations, recurrent gastrointestinal bleeding, severe anemia, and repeated endoscopic and surgical interventions. By integrating the age-based clinical course with endoscopic, magnetic resonance imaging, gross pathological, and histopathological findings, this report illustrates the limitations of repeated lesion-directed therapy in multifocal BRBNS. It also describes the patient's early clinical and hematologic course during 2 months of follow-up after transfusion, hematinic therapy, and lauromacrogol sclerotherapy of multiple soft-tissue lesions. The case therefore underscores the need for multidisciplinary assessment and longer-term evaluation of systemic therapy while avoiding inference of durable disease control from short-term improvement.

## 2 Case presentation

A 20-year-old Chinese man of Han ethnicity was admitted with recurrent melena and fatigue lasting for 2 months, against a background of multiple pale-blue superficial masses and a long-standing history of recurrent gastrointestinal bleeding since early childhood. Multiple pale-blue to bluish-purple masses involving the skin and underlying soft tissues were first noted at 18 months of age. The lesions were irregularly distributed, gradually increased in number, and subsequently involved multiple anatomical sites, including the left thigh and buttock, left knee, left elbow, posterior neck, and right shoulder.

At 7 years of age, persistent recurrent gastrointestinal bleeding prompted the first systematic gastrointestinal evaluation, which revealed multiple intestinal venous malformations and led to partial intestinal resection. Gross examination of the resected intestinal specimen showed multiple reddish-blue nodular lesions of varying sizes involving the intestinal wall. Microscopically, the lesions consisted of multiple irregular, markedly dilated, thin-walled vascular spaces beneath the intestinal mucosa and within the submucosa, with erythrocyte-filled lumina, consistent with venous malformation. On the basis of the cutaneous and soft-tissue lesions, multiple intestinal lesions, and postoperative pathological findings, the patient was diagnosed with BRBNS.

Other differential diagnoses were considered. Hereditary hemorrhagic telangiectasia was considered unlikely because the patient had no recurrent epistaxis, mucocutaneous telangiectasia, or relevant family history, and the morphology of the lesions was not typical of telangiectasia or arteriovenous malformation. Klippel-Trenaunay syndrome was excluded because there was no capillary malformation (port-wine stain), limb overgrowth or asymmetry, or prominent varicosities. Maffucci syndrome was excluded because no enchondromas or skeletal deformities were present. Proliferative vascular tumors, including infantile hemangioma, were not supported by histopathology, which showed dilated, thin-walled venous channels lined by flattened endothelial cells without cytological atypia, supporting a venous malformation rather than a vascular tumor. Taken together, the characteristic combination of multifocal cutaneous and soft-tissue venous malformations, gastrointestinal venous malformations, and recurrent bleeding supported the diagnosis of BRBNS.

Between 7 and 15 years of age, following the first systematic gastrointestinal evaluation and partial intestinal resection at 7 years of age, the patient continued to experience recurrent gastrointestinal bleeding, manifested as melena, hematochezia, and progressive anemia. During this period, he required multiple blood transfusions and underwent endoscopic ligation and further intestinal resections for bleeding control. Multiple superficial and soft-tissue masses involving the left thigh and buttock, left knee, left elbow, posterior neck, and right shoulder were also surgically excised. Although these lesion-directed interventions provided temporary symptomatic relief, gastrointestinal bleeding recurred during follow-up. Histopathological examination of the resected lesions demonstrated dilated, thin-walled venous channels lined by flattened endothelial cells without cytological atypia, consistent with venous malformations and further supporting the diagnosis of BRBNS.

**Figure 1:**
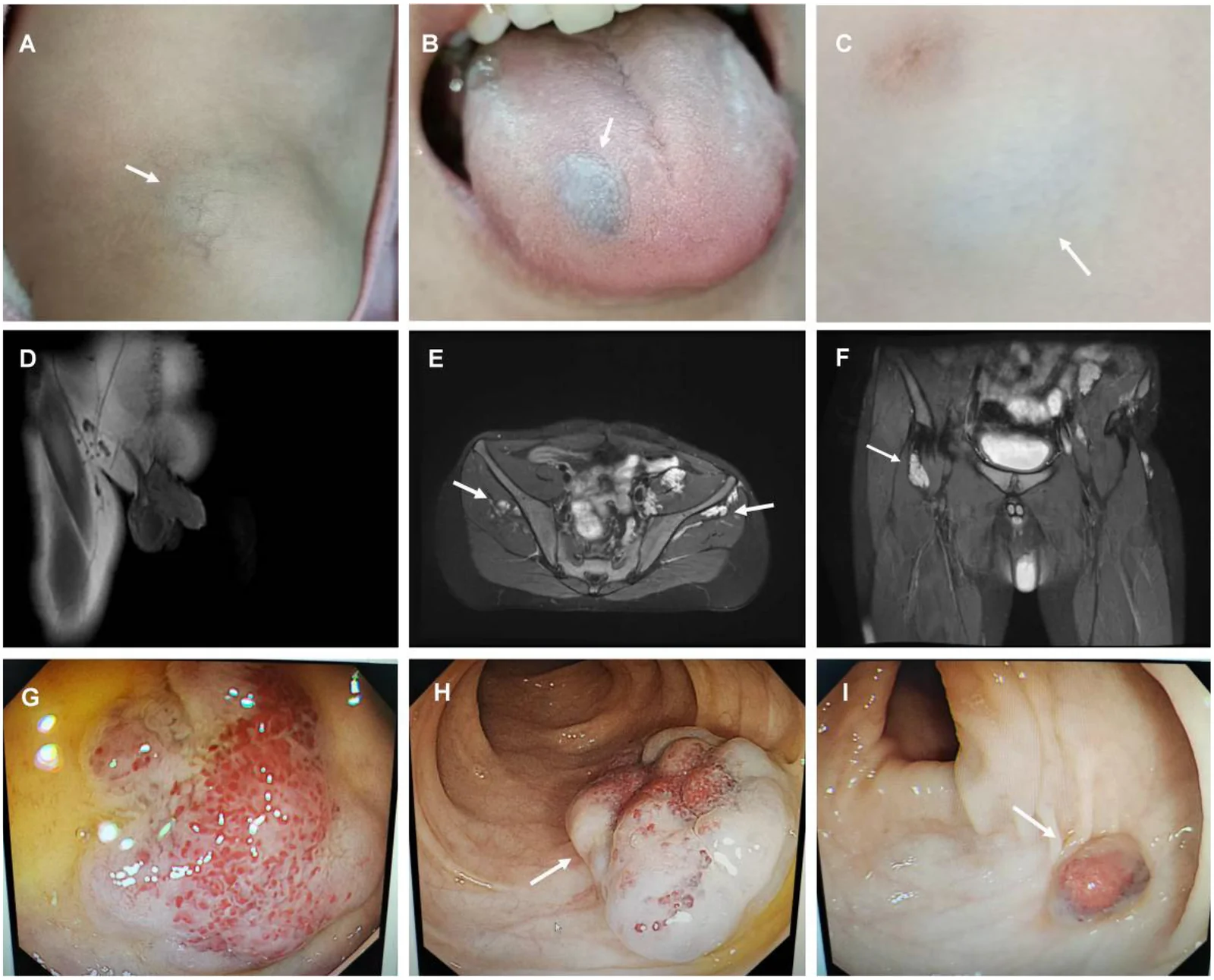
Clinical, magnetic resonance imaging, and endoscopic manifestations of multifocal venous malformations in blue rubber bleb nevus syndrome. **(A–C)**: Clinical photographs showing representative bluish superficial venous malformations involving the skin and tongue. **(D)**: Magnetic resonance imaging showing a large lobulated superficial soft-tissue venous malformation in the gluteal/proximal thigh region. **(E)**: Axial magnetic resonance image showing multiple venous malformations involving the pelvic and gluteal soft tissues. **(F)**: Coronal magnetic resonance image showing multifocal venous malformations involving the pelvic and proximal thigh soft tissues bilaterally. **(G)**: Gastroscopy showing a 0.3-cm mucosal venous malformation with superficial ectatic vessels along the greater curvature of the gastric body. **(H)**: Colonoscopy showing a protruding reddish-blue venous malformation in the ascending colon. **(I)**: Colonoscopy showing a similar venous malformation in the rectum. White arrows indicate representative lesions in panels A-C, E, F, H, and I. The endoscopic and magnetic resonance images in panels D-I were obtained during the hospitalization at 17 years of age.

**Figure 2:**
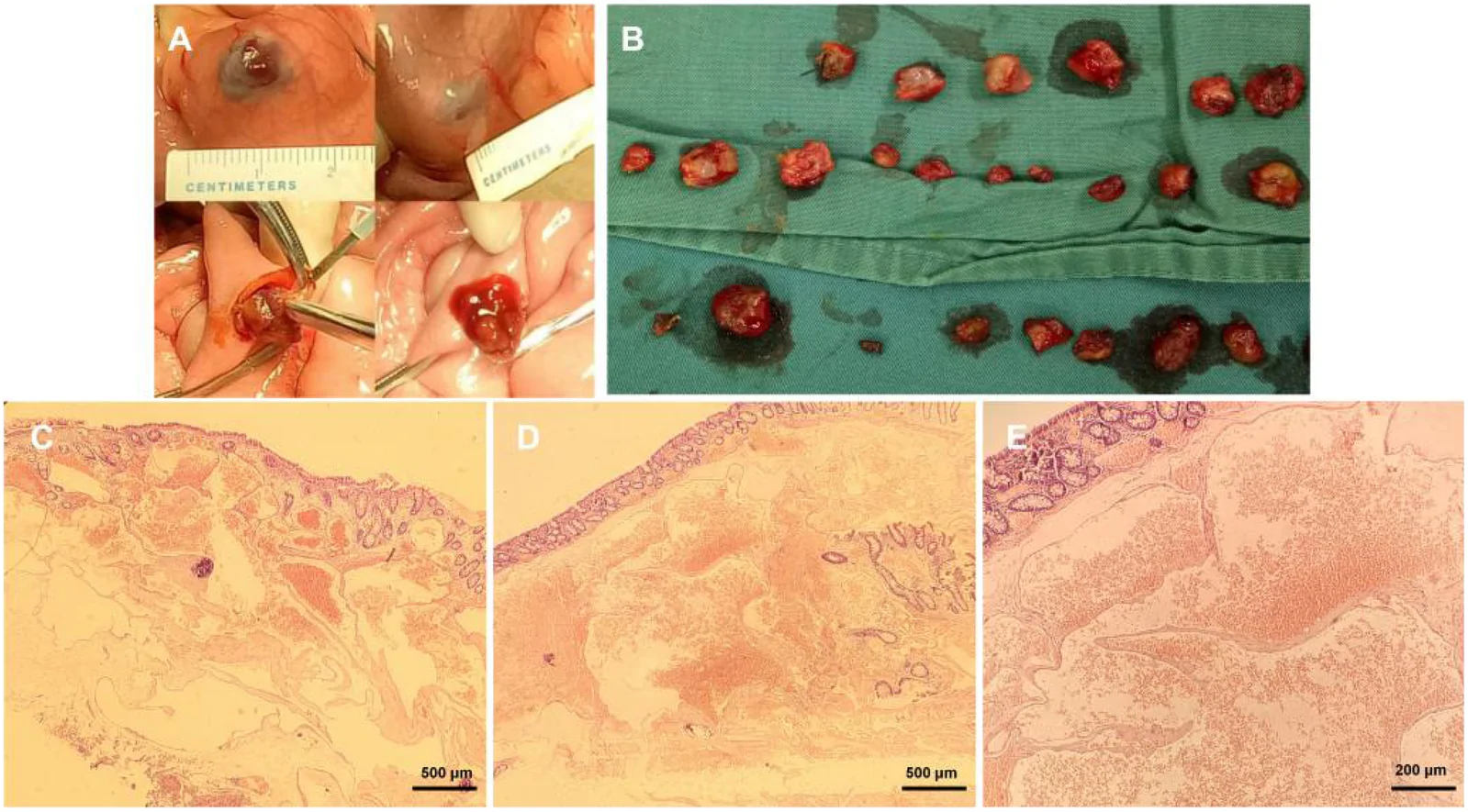
Intraoperative, gross, and histopathological findings of gastrointestinal venous malformations in BRBNS. **(A)**: Representative intraoperative views show multiple bluish-purple nodular lesions involving the intestinal wall, with ruler-assisted size assessment and surgical excision. **(B)**: Gross appearance of multiple excised reddish-blue nodular lesions of varying sizes. **(C,D)**: Low-power hematoxylin and eosin-stained sections demonstrate multiple irregular, markedly dilated, thin-walled vascular spaces beneath the intestinal mucosa and within the submucosa. **(E)**: Higher-power view shows erythrocyte-filled vascular lumina lined by flattened endothelial cells, consistent with venous malformation. Scale bars: 500 μm in panels C and D and 200 μm in panel E. The intraoperative, gross, and histopathological findings shown in panels A-E were obtained during the intestinal surgery performed at 17 years of age.

At 17 years of age, the patient was admitted with worsening hematochezia, palpitations, and fatigue. Laboratory examination revealed severe microcytic anemia, with a hemoglobin level of 34 g/L, hematocrit of 12.2%, and mean corpuscular volume of 56.3 fL. Gastroscopy identified venous malformations in the stomach and duodenum, while colonoscopy showed multiple lesions in the ascending colon, transverse colon, and rectum. Pelvic MRI demonstrated multifocal venous malformations involving the gluteal region, pelvis, and proximal thighs. Because of the severe anemia and extensive gastrointestinal bleeding, endoscopic sclerotherapy of the gastric and duodenal lesions was followed by open adhesiolysis and systematic exploration of the intestine. Multiple scattered intestinal venous malformations, including actively bleeding lesions, were individually excised using longitudinal incision and transverse closure. Recovery was uneventful, with normalization of bowel movements and improvement in bleeding symptoms and anemia. Representative clinical, magnetic resonance imaging, and endoscopic findings are shown in Figure 1, whereas the corresponding intraoperative, gross, and histopathological findings are shown in Figure 2.

At 20 years of age, the patient was admitted with recurrent melena and fatigue lasting for 2 months. On examination, he was conscious and alert but markedly anemic, with numerous pale-blue to bluish-purple superficial masses of varying sizes were irregularly distributed over the trunk and extremities; the larger lesions measured approximately 1.2 × 0.6 cm and were well defined and non-tender and multiple healed surgical scars on the chest wall, abdominal wall, and limbs. The abdomen was soft without tenderness or rebound tenderness, the liver and spleen were not palpable below the costal margins, and no lower-limb edema was present. His temperature was 36.6 degrees C, pulse rate 90 beats/min, respiratory rate 22 breaths/min, and blood pressure 131/59 mmHg. Complete blood count showed severe microcytic anemia, with a hemoglobin level of 55 g/L, hematocrit of 21.2%, and mean corpuscular volume of 58.7 fL. His parents were healthy, and he reported no family history of vascular malformations or related hereditary disorders. He had no known drug or food allergies, did not smoke or drink alcohol, and had no documented regular long-term medication use before admission. During the current hospitalization, management focused on correction of chronic blood-loss anemia and local treatment of the extensive symptomatic superficial and soft-tissue venous malformations. The patient received packed red-cell transfusion, oral hematinic and hemostatic therapy, and multiple sessions of percutaneous lauromacrogol sclerotherapy. Unlike the intervention performed at 17 years of age, no gastrointestinal endoscopic therapy or intestinal resection was undertaken during this admission.

At discharge, the patient was clinically stable, with hemoglobin increased to 98 g/L and no documented treatment-related complications. Follow-up assessments were performed at 2 weeks, 1 month, and 2 months after discharge. No recurrent melena or hematochezia, further transfusion requirement, or treatment-related complication was reported; oral iron supplementation was continued, and hemoglobin increased from 98 g/L at discharge to 113 g/L at 2 months. The patient’s age-based clinical course, diagnostic assessments, treatments, and outcomes are summarized in Table 1.

**Table 1:** Age-based clinical course, diagnostic assessment, treatment, and outcomes in the patient with BRBNS.

Patient age	Key clinical manifestations	Diagnostic assessment	Treatment	Pathological findings/outcome
18 months	Multiple bluish-purple cutaneous and soft-tissue venous malformations; progressive increase in number.	No systematic diagnostic assessment documented.	No treatment documented.	Lesions persisted and increased over time.
7 years	Gastrointestinal bleeding; multiple intestinal venous malformations	Clinical examination and postoperative histopathology.	Partial intestinal resection.	Clinical and pathological findings supported BRBNS.
9 years	Recurrent gastrointestinal bleeding.	Endoscopic evaluation of recurrent intestinal lesions.	Endoscopic lesion ligation.	Bleeding control was inadequate.
12 years	Recurrent melena.	Evaluation for recurrent intestinal bleeding.	Intestinal lesion resection and ligation.	Symptoms were temporarily relieved.
15 years	Hematochezia and anemia.	Evaluation at an outside hospital.	Blood transfusion; resection of small-intestinal and colonic lesions; excision of superficial lesions.	Pathology supported BRBNS-associated venous malformations; postoperative recovery was uneventful.
17 years	Worsening hematochezia, palpitations, fatigue, and severe microcytic anemia (hemoglobin, 34 g/L).	Blood count, gastroscopy, colonoscopy, and pelvic MRI.	Endoscopic sclerotherapy of gastric and duodenal lesions; open adhesiolysis and excision of multiple intestinal venous malformations.	Postoperative recovery was uneventful; bowel movements normalized, and bleeding symptoms and anemia improved.
20 years (current admission)	Recurrent melena (2 months), fatigue, extensive superficial and soft-tissue venous malformations, and severe microcytic anemia (hemoglobin, 55 g/L).	Admission vital signs, physical examination, and blood count.	Packed red-cell transfusion, oral hematinic and hemostatic therapy, and multiple sessions of percutaneous lauromacrogol sclerotherapy for symptomatic superficial and soft-tissue venous malformations.	Hemoglobin, 98 g/L at discharge; clinically stable; no inpatient treatment-related complication documented.

A schematic age-based overview of the patient’s clinical course, including major diagnostic findings, therapeutic interventions, and outcomes, is provided in Supplementary Data sheet 1.

## 3 Discussion

The principal educational value of this case lies in documenting a long-standing disease course from infancy to young adulthood, characterized by recurrent gastrointestinal bleeding since early childhood, repeated transfusion support, and multiple endoscopic and surgical interventions. It illustrates that lesion-directed therapy may provide temporary control but remain insufficient in multifocal BRBNS. BRBNS is a congenital venous malformation syndrome that can involve multiple organs, most commonly the skin and gastrointestinal tract (1, 2, 6). The disease is extremely rare, with only approximately 300 cases reported worldwide, and the estimated incidence is approximately 1 in 14,000 individuals (1). BRBNS usually manifests in early childhood, although some patients are diagnosed only in adulthood. The oldest confirmed patient reported in the literature was 89 years old, indicating that delayed diagnosis may occur even in very late adulthood (7, 8).

The fundamental pathological lesion of BRBNS is a venous malformation rather than a hemangioma in the conventional proliferative vascular-tumor sense (9–11). Histopathologically, lesions are typically composed of dilated, tortuous, thin-walled venous-like vascular channels lined by flattened endothelial cells, sometimes accompanied by thrombosis or calcification. These features are consistent with low-flow venous malformations and differ from proliferative vascular tumors such as infantile hemangioma. Accordingly, the International Society for the Study of Vascular Anomalies (ISSVA) classifies BRBNS within the spectrum of venous malformations on the basis of its clinical, pathological, and molecular features (9, 10). According to the 2025 ISSVA classification, BRBNS is categorized as a slow-flow vascular malformation within the multifocal venous malformations subgroup (12). This classification reinforces that BRBNS represents a systemic venous malformation rather than a vascular tumor.

The pathogenesis of BRBNS remains incompletely defined. Current evidence suggests that somatic mutations in TEK/TIE2 may contribute to disease development. TIE2 is a tyrosine kinase receptor involved in several stages of angiogenesis, and somatic activating mutations in TEK may cause persistent receptor activation and dysregulated vascular development (3, 13, 14). Soblet et al. identified somatic TEK/TIE2 mutations in 15 of 17 patients with BRBNS and detected somatic TEK mutations in 5 of 6 patients with sporadic multifocal venous malformations. Unlike common solitary venous malformations, which are usually associated with a single TEK mutation, BRBNS and some multifocal venous malformations more frequently harbor double-cis somatic mutations on the same allele. Identical mutations in different lesions from the same patient further suggest that multifocal lesions may share a common molecular basis. Functional experiments showed that the recurrent T1105N-T1106P mutation can induce ligand-independent activation of TIE2 and enhance endothelial cell survival, invasion, and clonogenic capacity. However, because available studies remain limited in sample size and TEK mutations have not been detected in all patients, TEK/TIE2 mutations should not be regarded as the sole cause of BRBNS (13).

BRBNS is easily overlooked or misdiagnosed, and no guideline has established a unified standard treatment strategy (6, 15). Therapeutic selection should consider lesion extent, bleeding severity, degree of anemia, lesion accessibility, patient age, and general condition. For patients with mild symptoms or without active bleeding, supportive management, including iron supplementation, anemia correction, and regular follow-up, may be sufficient (7). For localized bleeding lesions that are accessible endoscopically or surgically, endoscopic band ligation, sclerotherapy, resection, argon plasma coagulation, laser therapy, or surgery may be considered (4, 5, 16, 17).

Capsule endoscopy is a noninvasive and well-tolerated imaging modality that enables visualization of the entire small intestine and may facilitate the diagnosis and assessment of gastrointestinal involvement in BRBNS (2, 18, 19). Surgical experience reported by Fishman et al. suggests that, in selected patients with severe gastrointestinal bleeding, systematic localization and maximal removal of bleeding gastrointestinal lesions can markedly reduce long-term bleeding burden. However, this strategy is invasive and technically demanding; it is therefore more appropriate for patients with identifiable lesions and recurrent bleeding than as routine therapy for all patients with BRBNS (4).

The present patient represents a challenging form of BRBNS with extensive cutaneous and gastrointestinal involvement. His clinical course was marked by long-standing recurrent gastrointestinal bleeding since early childhood, repeated transfusion requirements, and relapse after multiple endoscopic and surgical interventions. Although lesion-directed procedures achieved temporary hemostasis, they did not provide sustained control of the multifocal disease. The repeated need for transfusion and reintervention therefore reflects the cumulative burden of diffuse BRBNS rather than the failure of any single procedure. Management goals should extend beyond short-term control of individual lesions to reducing overall bleeding, transfusion requirements, and subsequent interventions. In recent years, sirolimus, an mTOR inhibitor, has emerged as a promising systemic treatment option for patients with extensive, multifocal, recurrently bleeding BRBNS or for those who are unsuitable for repeated endoscopic or surgical intervention (15, 20). Yuksekkaya et al. reported that low-dose sirolimus reduced lesion size and controlled severe recurrent gastrointestinal bleeding without significant adverse effects during 20 months of follow-up. Prospective evidence also associated sirolimus with reduced venous malformation volume and gastrointestinal bleeding, increased hemoglobin levels, and lower transfusion requirements (21, 22). These findings suggest that sirolimus is among the most promising systemic therapeutic options for extensive or bleeding BRBNS that cannot be adequately controlled by conventional local therapies (23, 24). Nevertheless, the supporting evidence remains largely based on case reports, case series, and small prospective studies, and large-scale randomized controlled trials are still lacking. Whether sirolimus is effective in somatic mutation-driven BRBNS, and the extent of its benefit in such patients, also remain unclear. Multidisciplinary assessment and higher-quality studies are therefore needed to clarify its long-term efficacy and safety (25, 26). A comparison of representative BRBNS reports from the past 10 years and the present case, including clinical characteristics, treatment strategies, follow-up, and outcomes, is presented in Table 2. Compared with recent reports describing longer follow-up after sirolimus or combined endoscopic and surgical treatment, the present case documents the cumulative burden of a prolonged clinical course despite repeated lesion-directed interventions. However, because no systemic therapy was administered and follow-up was limited to 2 months, the observed short-term clinical and hematologic improvement should not be interpreted as durable disease control.

**Table 2:** Comparison of representative BRBNS reports and the present case.

Study and patient	Cutaneous/GI involvement	Anemia/transfusion	Imaging/genetics	Treatment	Follow-up/outcome
Zhou et al. (2021) (21); prospective study, *n* = 11 Median 14 y (range 5–49); 4 M/7 F	Multifocal cutaneous and GI VMs	Severe chronic anemia; transfusion-dependent at baseline	Serial whole-body MRI (0, 3, 6, and 12 mo); genetics NR	Oral sirolimus 1.0 mg/m^2^; trough 3–10 ng/mL	12 mo: lesion size decreased 13.0%; Hb increased; only 1 transfusion in the cohort; grade 1–2 adverse effects
Suarez et al. (2022) (5); case report 20 y/M	Blue nevi; gastric, duodenal, small-bowel, and colonic VMs; occult GI bleeding	Hb 1.7 g/dL; several packed-RBC units	EGD/colonoscopy, VCE, and endoscopic US; genetics NR	Iron; EMR/hybrid ESD; laparoscopic excision of 11 transmural lesions	7 mo: asymptomatic; no further transfusion/hospitalization; discharge Hb 10.8 g/dL
Duong et al. (2022) (22); case series, *n* = 4 Pediatric patients; individual ages/sex NR	GI VMs with bleeding; cutaneous distribution NR	Anemia; severity and transfusion burden NR	Imaging/genetics NR	Oral sirolimus	Successful GI bleeding control; individual follow-up/outcomes NR in the abstract
Samanta et al. (2023) (16); case 1 11 y/M	Toe/ankle papules; gastric, small-bowel, and colorectal VMs; occult GI bleeding	Hb 6.6 g/dL; transfusion NR	EGD/colonoscopy, VCE/deep enteroscopy; CT angiography/enterography negative; genetics NR	EBL plus propranolol	6 mo: Hb 11.2 g/dL; no further decrease
Samanta et al. (2023) (16); case 2 4 y/M	Multifocal skin papules; esophageal, gastric, jejunal, and colorectal VMs; hematochezia/melena	Transfusion every 3–6 mo for 2 y; Hb NR	EGD/colonoscopy and VCE; CT angiography/enterography negative; transient intussusception; genetics NR	EBL ×3 over 3 y plus propranolol; prior skin debulking	8 y: no overt GI bleeding; transfusion every 1–2 y; few new colonic lesions at 3 y
Liu et al. (2024) (26); case report 12 y/F	Purple-blue lip/skin nodules; gastric and intestinal VMs; occult GI bleeding	Hb 48 g/L; 7 transfusions/2 y plus 1 during treatment	MRI: 0.5–1.5-cm small-intestinal, colonic, and rectal lesions; genetics NR	Lauromacrogol sclerotherapy plus sirolimus 1.6 mg/m^2^/day, iron, and transfusion	1 y: Hb 140 g/L at 3 mo and 132 g/L at 1 y; no anemia/GI bleeding
Méndez Cordero et al. (2024) (27); case report; 14 y/M	Multifocal cutaneous venous malformations; gastric and multiple colonic lesions; intermittent bloody stools and positive fecal occult blood	Hb 6.3 g/dL; packed-RBC transfusion during admission	Dermoscopy, skin biopsy, EGD, and colonoscopy; genetic testing NR	Packed-RBC transfusion and supportive multidisciplinary management; previous propranolol was ineffective	Discharged after 14 days without complications; outpatient follow-up planned, but duration and subsequent outcome NR
Present case (2026) 20 y/M	Multifocal cutaneous/soft-tissue and GI VMs; recurrent melena/hematochezia	Hb 55 g/L at current admission; repeated prior transfusions; packed RBCs during current admission	Endoscopy and pelvic MRI at 17 years showed multifocal GI, gluteal, pelvic, and proximal-thigh VMs; no genetic testing	At 17 years: endoscopic sclerotherapy and open excision of multiple intestinal lesions; at 20 years: transfusion, hematinic/hemostatic therapy, and multiple sessions of percutaneous lauromacrogol sclerotherapy for superficial and soft-tissue lesions; no systemic therapy documented	2 mo: no recurrent GI bleeding or transfusion; Hb 113 g/L; no complications; durability uncertain

This report has several limitations. Genetic testing was not performed, and detailed records of some previous interventions conducted at outside institutions were unavailable. In addition, follow-up was limited to 2 months, and no systemic therapy was administered during the documented follow-up. Therefore, this case does not allow assessment of durable disease control or of the efficacy and safety of systemic agents such as sirolimus.

## 4 Conclusion

BRBNS is a multifocal venous malformation in which repeated lesion-directed interventions may achieve temporary hemostasis without sustained control of the overall disease burden. The long-term disease course in this patient underscores the need for multidisciplinary assessment, longitudinal surveillance, and consideration of systemic therapy in selected patients, while longer follow-up is required to determine whether disease control is durable.

## 5 Patient perspective

The patient considered the repeated surgical procedures and blood transfusions over the years to be burdensome but understood their necessity during episodes of severe gastrointestinal bleeding and anemia. He was encouraged by the clinical improvement following the current treatment and expressed willingness to continue regular follow-up to monitor for recurrent bleeding and anemia.

## Data Availability

The raw data supporting the conclusions of this article will be made available by the authors, without undue reservation.

## References

[1] Becq A Bisdorff A Riccioni ME Blaise S Mallet S Toth E. Blue rubber bleb nevus syndrome: a European multicenter cohort study. Dig Liver Dis. (2025) 57:603–8. 10.1016/j.dld.2024.10.001 39426903

[2] Xia H Wu J Huang Y. Blue rubber bleb nevus syndrome: a single-center case series in 12 years. Transl Pediatr. (2021) 10:2960–71. 10.21037/tp-21-238 34976762 PMC8649602

[3] Diociaiuti A Rotunno R Pisaneschi E Cesario C Carnevale C Condorelli AG. Clinical and molecular Spectrum of sporadic vascular malformations: a single-center study. Biomedicines. (2022) 10:1460. 10.3390/biomedicines10061460 35740480 PMC9220263

[4] Fishman SJ Smithers CJ Folkman J Lund DP Burrows PE Mulliken JB. Blue rubber bleb nevus syndrome: surgical eradication of gastrointestinal bleeding. Ann Surg. (2005) 241:523–8. 10.1097/01.sla.0000154689.85629.93 15729077 PMC1356993

[5] Suarez ZK Castaneda D Gonzalez A Castro FJ Erim T. Endoscopic and surgical management of blue rubber bleb nevus syndrome. ACG Case Rep J. (2022) 9:e00890. 10.14309/crj.0000000000000890 36277742 PMC9584184

[6] Kozai L Nishimura Y. Clinical characteristics of blue rubber bleb nevus syndrome in adults: systematic scoping review. Scand J Gastroenterol. (2023) 58:1108–14. 10.1080/00365521.2023.2214263 37211745

[7] Chen L-C Yeung C-Y Chang C-W Lee H-C Chan W-T Jiang C-B. Blue rubber bleb nevus syndrome (BRBNS): a rare cause of refractory Anemia in children. Children. (2022) 10:3. 10.3390/children10010003 36670554 PMC9856356

[8] Aron J Couturier A Sinayoko L Duedal V Ridel C Touzot M. An unusual cause of gastrointestinal bleeding in a hemodialysis patient. Hemodial Int. (2018) 22:E60–2. 10.1111/hdi.12657 29608808

[9] Wassef M Blei F Adams D Alomari A Baselga E Berenstein A. Vascular anomalies classification: recommendations from the international society for the study of vascular anomalies. Pediatrics. (2015) 136:e203–214. 10.1542/peds.2014-3673 26055853

[10] Wang MX Kamel S Elsayes KM Guillerman RP Habiba A Heng L. Vascular anomaly syndromes in the ISSVA classification system: imaging findings and role of interventional radiology in management. Radiogr: Rev Publ Radiol Soc N Am Inc. (2022) 42:1598–620. 10.1148/rg.210234 36190850

[11] Bakker AC Fishman SJ Liang MG Al-Ibraheemi A Kozakewich HP Mulliken JB. Immunohistochemical expression of lymphatic endothelial markers in blue rubber bleb nevus syndrome. Pediatr Dev Pathol: Off J Soc Pediatr Pathol Paediatr Pathol Soc. (2024) 27:228–34. 10.1177/10935266241228930 38512910

[12] Goldenberg DC Vikkula M Penington A Blei F Schultze-Kool L Wassef M. Updated classification of vascular anomalies: a living document from the international society for the study of vascular anomalies classification group. J Vasc Anom. (2025) 6:e113. 10.1097/JOVA.0000000000000113 PMC12237419 40636925

[13] Soblet J Kangas J Nätynki M Mendola A Helaers R Uebelhoer M. Blue rubber bleb nevus (BRBN) syndrome is caused by somatic TEK (TIE2) mutations. J Invest Dermatol. (2017) 137:207–16. 10.1016/j.jid.2016.07.034 27519652

[14] Xing Y Liu H Liu H Ding X Jing X. Genetic mutation and blue rubber bleb nevus syndrome: case reports and literature review. Front Genet. (2025) 16:1516562. 10.3389/fgene.2025.1516562 40584829 PMC12202426

[15] Rimondi A Sorge A Murino A Nandi N Scaramella L Vecchi M. Treatment options for gastrointestinal bleeding blue rubber bleb nevus syndrome: systematic review. Dig Endosc: Off J Jpn Gastroenterol Endosc Soc. (2024) 36:162–71. 10.1111/den.14564 PMC12136259 37029779

[16] Samanta A Poddar U Sarma MS Srivastava A Yachha SK Mohindra S. Endoscopic band ligation in blue rubber bleb nevus syndrome: a report of two children. JPGN Rep. (2023) 4:e344. 10.1097/PG9.0000000000000344 38034424 PMC10684117

[17] Unoura S Toya Y Kasugai S Kumei T Yamazato M Sasaki Y. Successful endoscopic sclerotherapy with bile duct stenting for a vascular malformation neighboring the duodenal papilla in blue rubber bleb nevus syndrome. DEN OPEN. (2022):2:e113. 10.1002/deo2.113 35873521 PMC9302048

[18] Reddy YK Bayoumi M Barnes M Gillespie W Kamal F Ismail M. An unusual and challenging cause of small bowel bleeding: isolated gastrointestinal blue rubber bleb nevus syndrome. Transl Gastroenterol Hepatol. (2022) 7:12. 10.21037/tgh.2020.02.19 35243121 PMC8826042

[19] Nitta K Matsui A Araki A Kikuchi D Hoteya S. Clipping with double-balloon endoscopy for small intestinal venous malformations in a patient with blue rubber bleb nevus syndrome. Clin J Gastroenterol. (2022) 15:901–6. 10.1007/s12328-022-01670-0 35864387

[20] Yuksekkaya H Ozbek O Keser M Toy H. Blue rubber bleb nevus syndrome: successful treatment with sirolimus. Pediatrics. (2012) 129:e1080–1084. 10.1542/peds.2010-3611 22392180

[21] Zhou J Zhao Z Sun T Liu W Yu Z Liu J. Efficacy and safety of sirolimus for blue rubber bleb nevus syndrome: a prospective study. Am J Gastroenterol. (2021) 116:1044–52. 10.14309/ajg.0000000000001117 33416235

[22] Duong JT Geddis A Carlberg K Rudzinski E Len M Zheng HB. Sirolimus for management of GI bleeding in blue rubber bleb nevus syndrome: a case series. Pediatr Blood Cancer. (2022) 69:e29970. 10.1002/pbc.29970 36094280

[23] Gonzalez-Magaña R García-Romero MT Durán-McKinster C. Blue rubber bleb nevus syndrome successfully treated with sirolimus: a report of five pediatric patients. Int J Dermatol. (2024) 63:1620–2. 10.1111/ijd.17217 38679758

[24] Zhang B Li L Zhang N Zhao M Liu Y Wei L. Efficacy and safety of sirolimus in the treatment of blue rubber bleb naevus syndrome in paediatric patients. Clin Exp Dermatol. (2020) 45:79–85. 10.1111/ced.14003 31074881

[25] Goyal A Kathuria S Dash A Meena SS Khunger N. Oral sirolimus in blue rubber bleb naevus syndrome: evidence of safety, efficacy, and improved quality of life. Indian J Dermatol Venereol Leprol. (2025) 19:1–3. 10.25259/IJDVL_248_2025 41100396

[26] Liu L Wang L Hu F. Case report: combination of sirolimus and endoscopic lauromacrogol sclerotherapy in the management of blue rubber bleb nevus syndrome with gastric tract bleeding. Front Pediatr. (2024) 12:1488466. 10.3389/fped.2024.1488466 39867693 PMC11757019

[27] Méndez Cordero PD Mariscal Garcia RS Montalván Durazno BM Oliveros Rivero JA Cornejo Baldeón AL. El enigma del síndrome de bean: un caso clínico que desafía lo conocido. Cienc Lat Rev Cient Multidiscip. (2024) 8:11862–74. 10.37811/cl_rcm.v8i6.16012

