# Supplementary material for: When lesion-directed therapy is not enough: blue rubber bleb nevus syndrome in a young adult—a case report
Source: Front Pediatr. 2026 Sep 10;14:1916054. doi: 10.3389/fped.2026.1916054 (PMC13601257; doi:10.3389/fped.2026.1916054)
Supplement: Supplementary file 1 [file Datasheet1.pdf]

Longitudinal clinical course of a patient with blue rubber bleb nevus syndrome

18 months

PRESENTATION
Bluish-purple cutaneous and soft-tissue venous malformations

ASSESSMENT
Detailed assessment records unavailable

TREATMENT
Treatment records unavailable

OUTCOME
Lesions gradually increased

7 years

PRESENTATION
Gastrointestinal bleeding; multiple intestinal lesions

ASSESSMENT
Clinical evaluation and postoperative pathology

TREATMENT
Partial intestinal resection

OUTCOME
Pathology supported BRBNS

9–12 years

PRESENTATION
Recurrent gastrointestinal bleeding, including melena

ASSESSMENT
Repeated endoscopic evaluation

TREATMENT
Endoscopic ligation and intestinal resection

OUTCOME
Temporary or inadequate control

15 years

PRESENTATION
Hematochezia and anemia

ASSESSMENT
Evaluation at an outside hospital

TREATMENT
Blood transfusion; resection of small-intestinal, colonic, and superficial lesions

OUTCOME
Uneventful postoperative recovery

17 years

PRESENTATION
Worsening hematochezia, palpitations, and fatigue; Hb 34 g/L

ASSESSMENT
CBC, gastroscopy, colonoscopy, and pelvic MRI

TREATMENT
Endoscopic sclerotherapy of gastric and duodenal lesions; open excision of multiple intestinal venous malformations

OUTCOME
Uneventful recovery; gastrointestinal bleeding and anemia improved

20 years

PRESENTATION
Recurrent melena and fatigue for 2 months; extensive superficial and soft-tissue venous malformations; Hb 55 g/L

ASSESSMENT
Vital signs, physical examination, and CBC

TREATMENT
Packed red-cell transfusion, oral hematinic and hemostatic therapy, and percutaneous lauromacrogol sclerotherapy

OUTCOME
Hb 98 g/L at discharge; clinically stable; no documented complications

---

FOLLOW-UP AFTER DISCHARGE (AGE 20 YEARS)

Early clinical and hematologic improvement;

longer follow-up is required to determine whether disease control is durable.

2 weeks
No recurrent melena or hematochezia; no transfusion

1 month
No recurrent bleeding; continued oral iron supplementation

2 months
No recurrent bleeding or further transfusion; Hb 113 g/L; no complications
